# Journalists as partners in early response to trauma: agreements, tensions, and future directions to aid collaboration

**DOI:** 10.3402/ejpt.v6.28544

**Published:** 2015-06-11

**Authors:** Elana Newman, Susan Drevo

**Affiliations:** 1 Dart Center for Journalism and Trauma Research Office, New York, NY, USA; 2 The University of Tulsa Institute for Trauma, Adversity and Injustice, The University of Tulsa, Tulsa, OK, USA

Implementing and understanding effective early response to trauma requires multidisciplinary collaboration. While interprofessional collaboration is a fundamental competency for disaster and trauma mental health, journalists are often overlooked professional responders. As a result, few professions receive training to effectively cooperate with journalists. Yet, journalists play key roles in trauma responses as professional fact-finders, image collectors, and narrators to bear witness and serve as the public's eyes and ears (Newman, Shapiro, & Nelson, 2009). Often, though not by design, news can function as a means of emergency communication, conveying information about safety, transportation routes, and volunteer needs to communities under threat. Such information can prevent non-essential personnel from congregating at disaster and crime scenes and interfering with the work of emergency responders. Conveying survivors’ perspectives and needs to government and agencies, and to the wider public, may leverage and facilitate the necessary mobilization of resources in response to catastrophe or crime. Journalists can help connect survivors with one another and their families. Rape survivors and survivors of torture, for example, can be empowered by news reports of others who have survived, bolstering their sense of hope and learning of resources. Investigative reports on the failures of systems to adequately prevent or respond to traumas in the community (e.g., child abuse and sexual violence) can serve as an impetus for the public to better understand these situations and intervene as citizens and neighbors.

However, the presence of journalists can intensify the existing sense of anxiety, anger, chaos, and burden for local leaders and communities who are struggling to address these complex issues. Clearly, concerns arise about interviews with survivors that seem insensitive or unethical. Advocates may fear that survivors are less likely to come forward to report crimes if they are publically identified. Interprofessional conflict and mistrust may also be perpetuated when simplified and inaccurate media portrayals of the aftermath of trauma overshadow nuanced coverage of events and shape distorted community perceptions (Newman & Shapiro, 2014). Thus, there are also fears and concerns about news practice. Given the distinct roles of journalists in disaster and crime response, greater understanding of journalists’ roles, ethics, professional identities, and culture is warranted. This presentation will articulate basic knowledge, skills, and attitudes that mental health professionals need to consider when working with journalists in the early response to trauma. The need for trust among emergency managers and journalists has been documented (McLean & Power, 2014), and this presentation will focus on strategies to create trust between mental health professionals and journalists.

Further, agreements, controversies, evidence, best practice, and gaps in knowledge to facilitate effective collaborations and consultations with journalists will be reviewed. For example, mental health professionals and journalists each have ethical codes that share values about minimizing harm, accountability, and truthfulness (e.g., American Psychological Association, 2010; Reuters, 2008; Society of Professional Journalists, 1996). However, journalists have historically exercised different methods and values for pursuing objectivity than clinicians or mental health researchers (Newman & Shapiro, 2014). Journalists, like clinicians, value the role of privacy for private citizens who are not public officials or those seeking influence, power, or attention. Although, unlike clinicians, journalists believe that overriding public needs, the public's right to know, and democracy's need for representative storytelling must be considered in the balance (Newman & Shapiro, 2014).

The small evidence base from those who have been covered in the news will be reviewed. For example, a qualitative study from Sweden suggests that survivors of train accidents may experience interacting with journalists and the news coverage itself as helpful, harmful, or neutral (Englund, Forsberg, & Saverman, 2014). Discussions of ways that journalists, clinicians, and researchers may foster positive outcomes for survivors will be discussed. Ultimately, this presentation aims to foster conversations about ways that trauma researchers and clinicians can ethically and effectively collaborate with journalists with understanding and respect for the aims, culture, and ethics of journalism.

This conference was funded by a grant from The Swedish Foundation for Humanities and Social Sciences (F14-1747:1).

## References

[CIT0001] American Psychological Association (2010). Ethical principles of psychologists and code of conduct.

[CIT0002] Englund L, Forsberg R, Saverman B.-I (2014). Survivors’ experiences of media coverage after traumatic injury events. International Emergency Nursing.

[CIT0003] McLean H, Power M. R (2014). When minutes count: Tension and trust in the relationship between emergency managers and the media. Journalism.

[CIT0004] Newman E, Shapiro B (2014). Clinicians and journalists responding to disaster. Journal of Child and Adolescent Psychopharmacology.

[CIT0005] Newman E, Shapiro B, Nelson S, Neria Y, Galea S, Norris F (2009). Journalism and media during disasters. Mental Health Consequences of Disaster.

[CIT0006] Reuters (2008). Handbook of journalism.

[CIT0007] Society of Professional Journalists (1996). Code of ethics.

